# SLC3A2 is a novel endoplasmic reticulum stress-related signaling protein that regulates the unfolded protein response and apoptosis

**DOI:** 10.1371/journal.pone.0208993

**Published:** 2018-12-28

**Authors:** Chunlei Liu, Xin Li, Chen Li, Zeyu Zhang, XiaoJian Gao, Zhilong Jia, HaiXu Chen, Qian Jia, Xiaojing Zhao, Jixuan Liu, Bohan Liu, Zhenguo Xu, Yaping Tian, Kunlun He

**Affiliations:** 1 Department of Translational Medicine, The General Hospital of Chinese People’s Liberation, Beijing, China; 2 Beijing Key Laboratory of Chronic Heart Failure Precision Medicine, Beijing, China; Duke University School of Medicine, UNITED STATES

## Abstract

Endoplasmic reticulum (ER) stress results from imbalances in unfolded/misfolded proteins, contributing to a wide variety of human diseases. To better understand the mechanisms involved in the cellular response to ER stress in cardiomyocytes, we previously conducted a genome-wide screening in an *in vitro* ER stress model of rat cardiomyocytes, which highlighted amino acid transporter heavy chain, member 2 (SLC3A2) as an important factor in ER stress. In the present study, we characterized the role of SLC3A2 during the unfolded protein response (UPR), as one of the primary pathways activated during ER stress. First, we confirmed the induction of *Slc3a2* mRNA expression following treatment with various ER stress inducers in rat cardiomyocytes (H9C2) and neural cells (PC12). Knockdown of *Slc3a2* expression with small interfering RNA (siRNA) revealed that the encoded protein functions upstream of three important UPR proteins: ATF4, ATF6, and XBP1. siRNA-mediated knockdown of both SLC3A2 and mammalian target of rapamycin 1 (mTOR1) revealed that mTOR1 acts as a mediator between SLC3A2 and the UPR. RNA sequencing was then performed to gain a more thorough understanding of the function of SLC3A2, which identified 23 highly differentially regulated genes between the control and knockdown cell lines, which were related to the UPR and amino acid transport. Notably, flow cytometry further showed that SLC3A2 inhibition also enhanced the apoptosis of rat cardiomyocytes. Taken together, these results highlight SLC3A2 as a complex, multifunctional signaling protein that acts upstream of well-known UPR proteins with anti-apoptotic properties, suggesting its potential as a therapeutic target for ER stress-related diseases.

## Introduction

Endoplasmic reticulum (ER) stress is involved in the development and pathology of various human diseases, including neurodegeneration, type 2 diabetes mellitus, Alzheimer’s disease, and cardiovascular disease. In eukaryotic cells, the ER is the primary site of protein biosynthesis as well as the early maturation steps for various proteins in the secretory pathway. This includes the folding of newly synthesized polypeptide chains and the addition of post-translational modifications that are essential for protein function. In fact, most nascent polypeptides are translocated in an unfolded state to the ER lumen, where they are then processed for folding. The balance between this inflow of unfolded proteins and the folding capacity of the ER is critical for cellular health [1, 2]. However, when ER function is disrupted and the inflow of unfolded proteins exceeds its processing capabilities, ER stress occurs. This stress in turn leads to the activation of a series of adaptive pathways known as the unfolded protein response (UPR), which is an attempt to maintain ER homeostasis. The molecular pathways defining UPR induction have been well characterized, mainly involving the activation of several key transcription factors, including activating transcription factor (ATF)4, ATF6, and X box-binding protein 1 (XBP1) [3,4]. These transcription factors subsequently enhance the synthesis of proteins involved in protein stabilization, ER-associated degradation, and other pathways that reduce the ER load. Unfortunately, ER dysfunction also affects many other aspects of cell physiology and secretion, and accumulation of unfolded or misfolded proteins resistant to proteasomal degradation in the ER can completely disrupt cellular function, resulting in apoptosis. Despite extensive research on these processes, several aspects of the mechanism underlying the cellular response to ER stress and the UPR remain unknown. Although the precise mechanisms remain poorly understood, several studies have shown that many of the genes highly expressed during ER stress are related to amino acid transporter heavy chain, member 2 (SLC3A2) signaling [5–8]. Indeed, our previous study using a tunicamycin (TM)-induced rat cardiomyocyte model of ER stress showed a time-dependent increase in the expression of 11 ER stress-related genes, including *Slc3a2* as the most highly expressed overall [9]. SLC3A2 is a transmembrane cell-surface protein of the solute carrier family, which plays a role in the transport of L-type amino acids and in the regulation of intracellular calcium [10,11]. However, the localization and underlying mechanism of action of this protein during ER stress have not been fully elucidated.

Thus, in the present study, we further explored the role of SLC3A2 in this response by determining its change in expression under stimulation of ER stress factors in rat cardiomyocytes and neural cells. We investigated the specific roles of SLC3A2, ATF4, and XBP1 in ER stress and the UPR by inhibiting their expression in rat cardiomyocytes through transfection of specific small interfering RNA (siRNA). We further examined the influence of SLC3A2 inhibition on apoptosis and the expression of UPR-related genes. In addition, LAPTM4b was reported to recruit LAT1-4F2hc (SLC7A5-SLAC3A2) to the lysosomes, leading to the uptake of Leu, and is required for mammalian target of rapamycin (mTOR1) activation [12]. Thus, to further characterize how SLC3A2 regulates the ER stress response, we examined the potential involvement of mTOR1. Finally, RNA-sequencing (RNA-seq) analysis was performed to determine the gene profile under SLC3A2 inhibition to further identify its general function. These results are expected to provide further insight into the molecular mechanism regulating the ER stress response, which can highlight targets for the diagnosis, treatment, and prevention of related diseases.

## Materials and methods

### Cell culture and treatments

H9C2 cells (rat cardiomyocytes) were purchased from the cell bank of Peking Union Medical College Hospital (Beijing, China) and were grown in Dulbecco’s modified Eagle’s Medium (DMEM) (Gibco) supplemented with 10% fetal bovine serum (FBS). PC12 cells (rat neuronal cells) were grown in DMEM supplemented with 10% FBS and 5% horse serum. The cells were transfected with the following rat-specific siRNAs (Sangon Biotech) using Lipofectamine RNAi MAX reagent (Invitrogen) according to the manufacturer’s protocol: *Slc3a2* sense GGACCUCACUCCCAACUAUTT, antisense AUAGUUGGGGAGUGAGGUCCTT; *Atf4* sense GCUGCUUUAUAUUUUACUUCUAATT, antisense UUAGAGUAAUAUAAGCAGCTT; *Atf6* sense GCAGUCGAUUAUCAUAUATT, antisense UAUACUGAUAAUCGACUGCTT; and *Xbp1* sense CAAGCUGGAAGCCAUUAAUTT, antisense AUUAAUGGCUUCCAGCUUGTT. ER stress and the consequent UPR was induced in H9C2 cardiomyocytes and PC12 neuronal cells with 10 μM TM (an N-linked glycosylation inhibitor), 5 μM thapsigargin (TG; a sarco-endoplasmic Ca^2+^-ATPase inhibitor), and 1 mM dithiothreitol (DTT; reduces disulfide linkages). Apoptosis was induced with 5 μM TG for 24 h.

### Immunofluorescence

For SLC3A2 and ATF4 immunostaining, cells were grown on glass coverslips, fixed with formalin, and treated with primary antibodies against SLC3A2 (Santa Cruz Biotechnology, sc-390154) and ATF4 (Abcam, ab194909) diluted in phosphate-buffered saline; 5% bovine serum albumin was added to block non-specific reactions. Following washing and incubation with the corresponding secondary antibody, nuclei were counterstained with 4′,6-diamidino-2-phenylindole. Immunofluorescence was visualized using a Leica fluorescence microscope.

### Reverse-transcription-quantitative polymerase chain reaction (RT-qPCR)

RNA was isolated using RNeasy columns (Qiagen) per the manufacturer instructions. In brief, the cells (1 × 10^7^) were lysed with Qiazol Lysis Reagent (Qiagen). The tube was then placed at room temperature for 5 min, and the sample was vortexed with 140 μL chloroform for 15 s, followed by centrifugation for 15 min at 12,000 ×*g* at 4°C. The upper fraction was transferred to a new tube, and a 1.5-volume of ethanol was added. After mixing, 700 μL of the sample was transferred to an RNeasy Mini spin column and centrifuged for 1 min at 8,000 ×*g*. The sample was transferred to a new 1.5-mL collection tube and 30 μL RNase-free water was added, followed by centrifugation for 1 min at 8,000 ×*g* to obtain the RNA as the pellet. The RNA quality was assessed using an Agilent Bioanalyzer Nano RNA Chip based on a 260/280 nm absorbance ratio of 1.8–2.0. High-quality RNA was then reverse-transcribed to cDNA with SuperScript III (Invitrogen), and the mRNA expression levels were assessed using the cDNA as a template using SYBR Green with the following primers: β-actin (forward: TAAAGACCTCTATGCCAACACAGT, reverse: CACGATGGAGGGGCCGGACTCATC); *Slc3a2* (forward: ACTTGGCTGAGTGGCAGAAT, reverse: AGATCGCTGGTGGATTCAAG); ATF4 (forward: CATTCCTCGATTCCAGCAAAGCAC, reverse: TTCTCCAACATCCAATCTGTCCCG); *Atf6* (forward: AGAGAAGCCTGTCACTGGTC, reverse: TAATCGACTGCTGCTTTGCC); and spliced *Xbp1* (forward: CCGCAGCAGGTGCAGG, reverse: GAGTCAATACCGCCAGAATCCA). The relative mRNA expression level of each target gene was normalized to that of β-actin, and calculated using the 2^–ΔΔCt^ method.

### Flow cytometry analysis of apoptosis

Apoptotic cells were identified using an Annexin V-fluorescein isothiocyanate (FITC)/propidium iodide (PI) apoptosis detection kit (BD Biosciences Pharmingen) according to the manufacturer’s protocol. In brief, the cells were suspended in 100 μL of binding buffer and stained with 5 μL of FITC-conjugated Annexin V and 5 μL PI for 15 min at room temperature in the dark. Samples were analyzed with a C6 flow cytometer (BD Biosciences), and the data were analyzed quantitatively with FlowJo software (Version 7.6.5).

### RNA-seq and data analysis

We used RNA-seq to identify other downstream targets of SLC3A2, using three treatment groups: non-treated control cells, cells transfected with scrambled siRNA and exposed to TG to induce ER stress, and cells transfected with *Slc3a2* siRNA and exposed to TG. Samples with an RNA integrity number >8 were considered to be of suitably high quality for RNA-seq. Sequencing libraries were prepared using an Illumina TruSeq Stranded Total RNA kit with Ribo-Zero Gold rRNA depletion and were sequenced on an Illumina HiSeq 2000 (paired-end, 100 base pairs, >50 M reads per sample; Rapid Run v2 flow cell).

Raw read data in fastq format were first processed using in-house Perl scripts. Adaptor sequences and low-quality reads were removed. The raw sequences were further processed to obtain clean reads, which were mapped to the reference genome sequence. Only reads with one or fewer mismatches were further analyzed and were annotated based on the reference genome. Prior to differential gene expression analysis, the read counts for each sequence library were adjusted with the edgeR program package using a scaling factor. Differential expression was analyzed using the DEG seq R package according to false discovery rate-adjusted *p*-values (q-values) were used: a gene was considered to be differentially expressed when q < 0.005 and |log2(fold change)| ≥ 1. Heatmaps were generated using the HemI 1.0—Heatmap illustrator. Samples were clustered using complete linkage and Pearson’s correlation. The DAVID (https://david.ncifcrf.gov) tool was used for gene ontology (GO) enrichment analysis and Kyoto Encyclopedia of Genes and Genomes (KEGG) pathway annotation [13,14] of the identified differentially expressed genes.

### Statistical analysis

All data, except for the RNA-seq data, are expressed as the mean ± standard deviation. One-way analysis of variance was used for multiple comparisons. Differences were considered significant at a *p* < 0.05. All experiments were replicated at least three times.

## Results

### ER stress activators induce SLC3A2 expression

**Fig 1:**
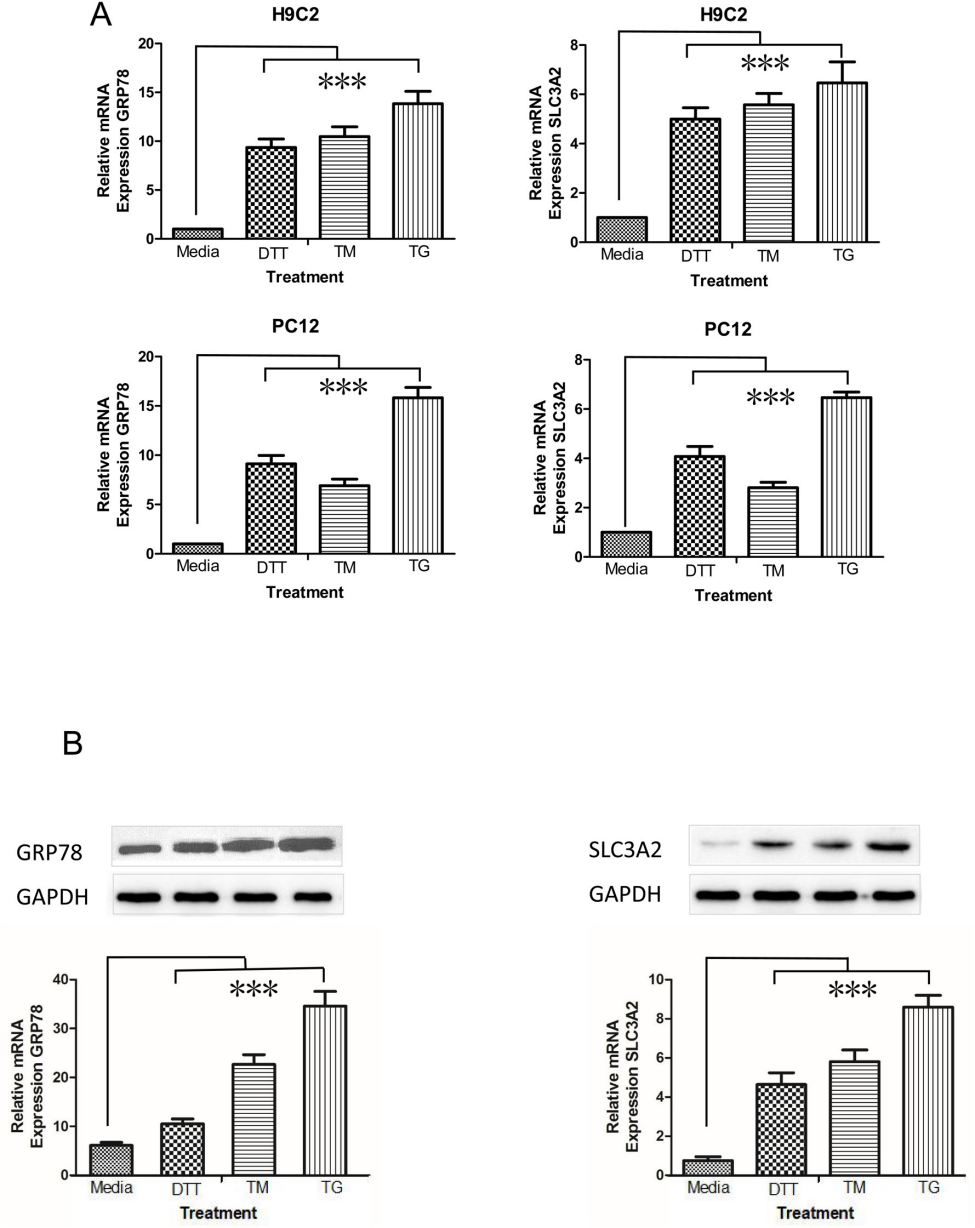
SLC3A2 is induced by ER stressors. SLC3A2 and GRP78 (A) mRNA and (B) expression as assessed by RT-qPCR and western blot analysis, respectively, in H9C2 and PC12 cells treated with DTT, TM, or TG for 4 h and in non-treated cells as controls. ***p < 0.001.

After induction of ER stress in H9C2 cardiomyocytes and PC12 neuronal cells with TM, TG, and DTT [15]. RT-qPCR and western blotting confirmed that SLC3A2 gene and protein expression was robustly induced (Fig 1A). Similar expression trends were observed for GRP78, a UPR marker, confirming the induction of ER stress and the UPR (Fig 1B). These data indicated that SLC3A2 is a novel member of the ER stress response.

### Subcellular localization of SLC3A2

**Fig 2:**
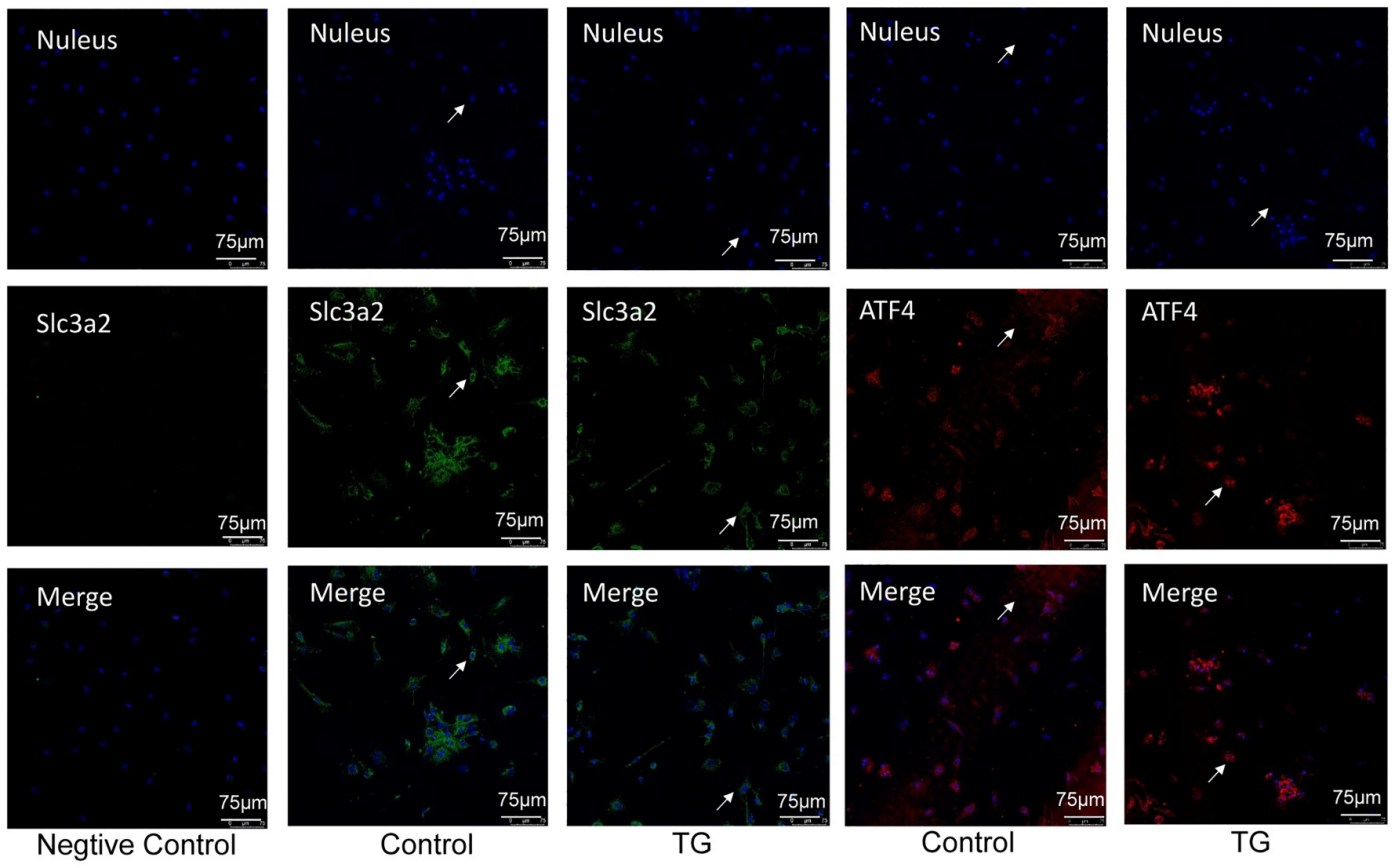
Subcellular localization of SLC3A2 in H9C2 cells is not affected by ER stress. Cells were immunostained with anti-SLC3A2 and anti-ATF4 antibodies, and nuclei were counterstained with DAPI. The fluorescence intensity was determined by confocal microscopy after 4 h of exposure to dimethyl sulfoxide (DMSO; control) or TG. Staining with or without SLC3A2 primary antibodies was performed as a positive or negative control.

SLC3A2 is a transmembrane glycoprotein that is primarily localized to the plasma membrane. Immunostaining in H9C2 cells revealed that SLC3A2 is primarily localized in the cytosolic compartment rather than the nucleus, similar to ATF4 (Fig 2). Next, we examined whether TG treatment would affect SLC3A2 and ATF4 localization. While ATF4 was translocated to the nucleus, SLC3A2 remained distributed in the cytosol upon TG treatment, indicating that the location of SLC3A2 protein is not affected by ER stress.

### SLC3A2 acts upstream of the UPR

**Fig 3:**
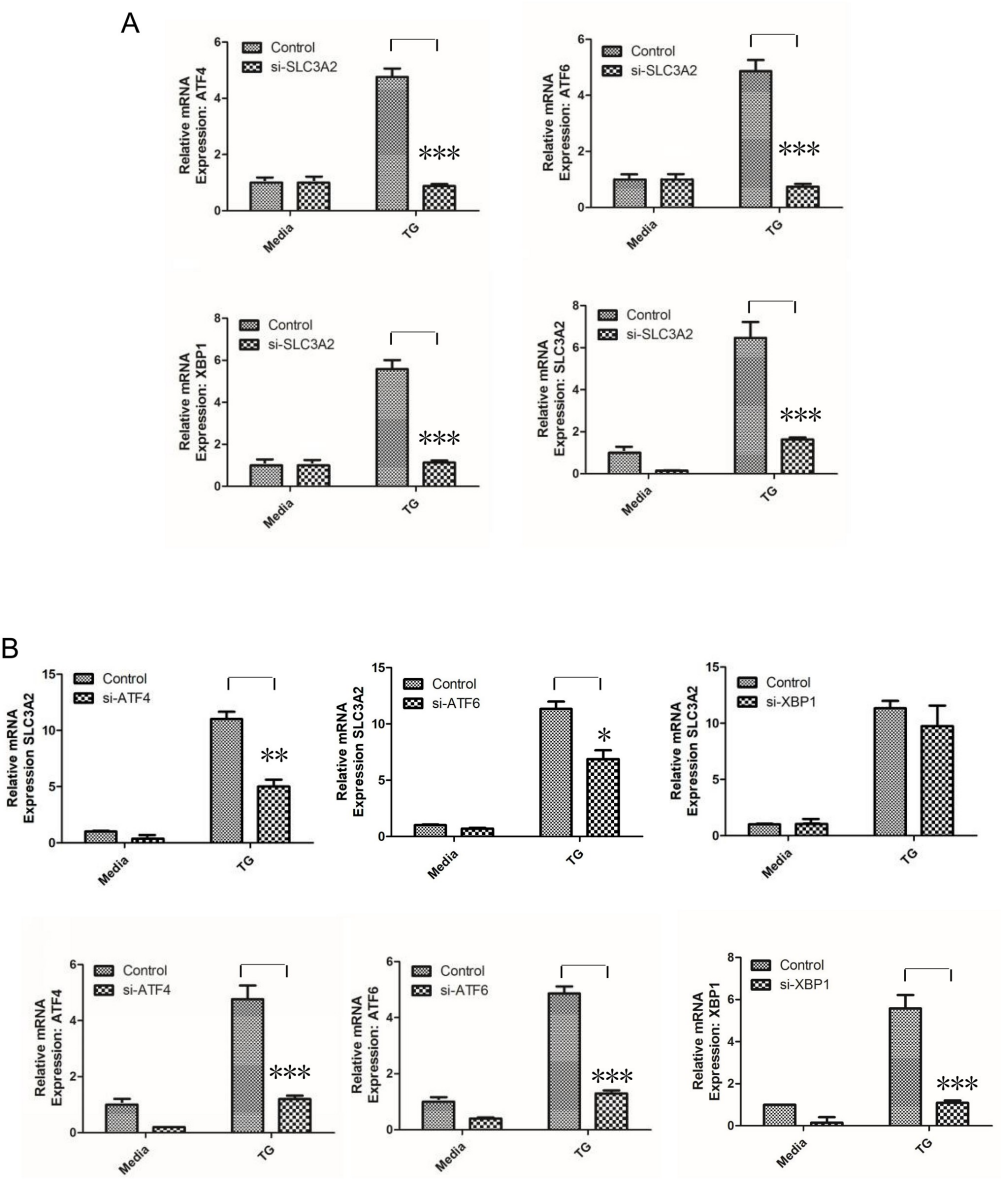
SLC3A2 knockdown inhibits *Atf4*, *Atf6*, and *Xbp1* mRNA expression following ER stress. (A) *Atf4*, *Atf6*, *Xbp1*, and *Slc3a2* mRNA levels were measured by RT-qPCR in TG-treated and non-treated H9C2 cells pretreated with *Slc3a2-*siRNA or scrambled control. Target mRNA levels were normalized to β-actin expression. ***p < 0.001. (B) *Slc3a2* mRNA levels were measured by RT-qPCR in TG-treated and non-treated H9C2 cells pretreated with siRNAs targeting *Atf4*, *Atf6*, or *Xbp1*, or scrambled control. *Slc3a2* mRNA expression was normalized to β-actin expression. **p < 0.01, *p < 0.05.

To characterize the signal transduction pathways affected by SLC3A2 upregulation, we examined the effect of siRNA-mediated *Slc3a2* knockdown on various UPR genes. *Slc3a2* knockdown inhibited the expression of the downstream genes *Atf4*, *Atf6*, and *Xbp1* (Fig 3A), indicating that the UPR is largely disrupted when SLC3A2 is inhibited. To validate the position of *Slc3a2* upstream of *Atf4*, *Atf6*, and *Xbp1*, the corresponding genes were individually knocked down with siRNA. *Xbp1* knockdown did not affect the response of *Slc3a2* expression following TG stimulation. As an activating transcription factor inhibiting the translocation of other factors, including ATF4, *Atf6* knockdown prevented the increase in *Slc3a2* expression following TG stimulation (Fig 3B).

### mTOR functions as a mediator between SLC3A2 and the UPR

**Fig 4:**
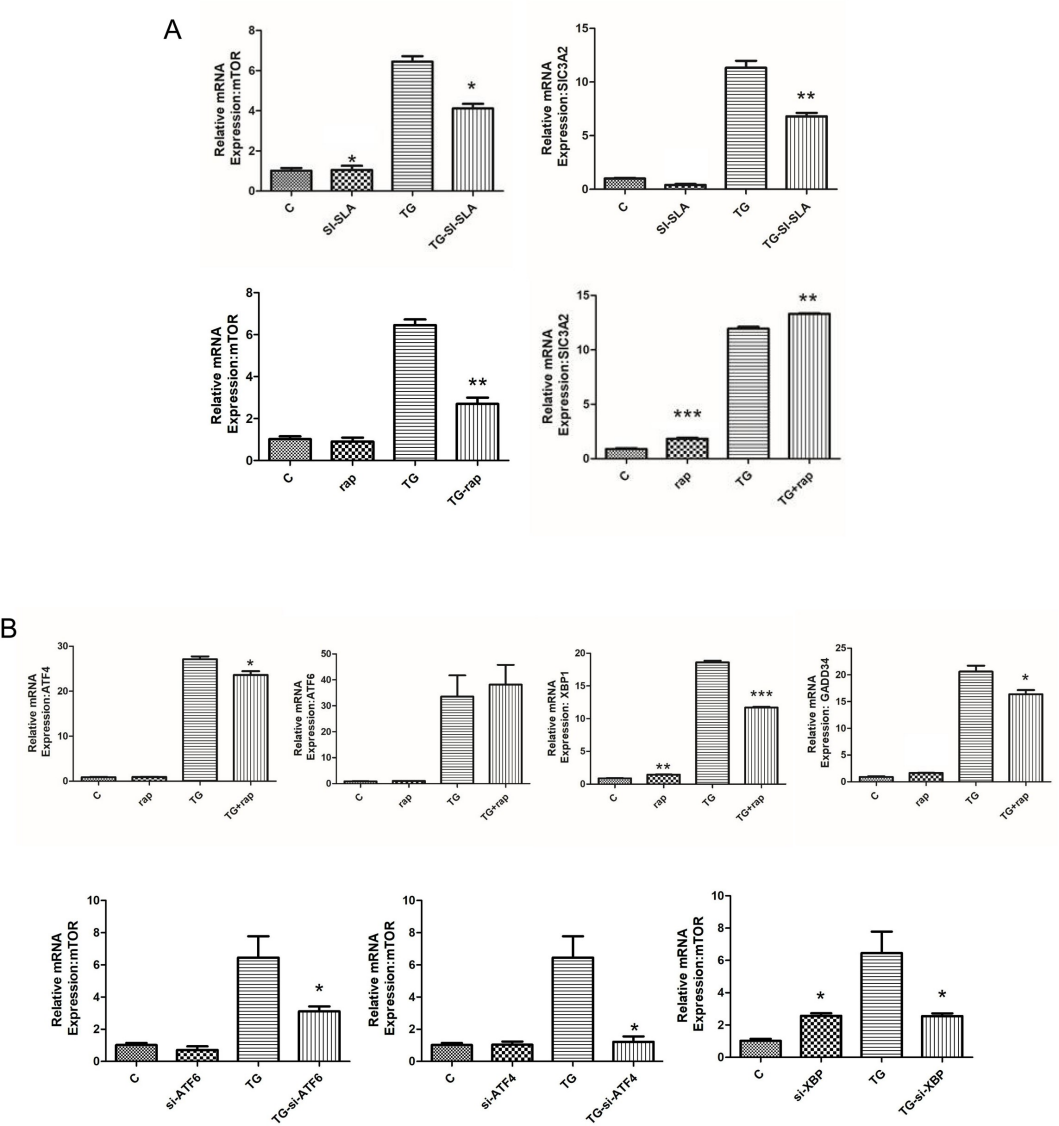
SLC3A2 knockdown inhibits mTOR1, and mTOR1 interacts with the UPR. *Atf4*, *Atf6*, *Xbp1*, and *Slc3a2* mRNA levels were measured by RT-qPCR in TG-treated and non-treated H9C2 cells pretreated with rapamycin, Slc3a2-siRNA, or scrambled control siRNA. Target mRNA levels were normalized to β-actin expression. ***p < 0.001. mTOR1 mRNA levels were measured by RT-qPCR in TG-treated and non-treated H9C2 cells that were pretreated with siRNAs targeting *Slc3a2*, *Atf4*, *Atf6*, or *Xbp1*, or scrambled control. Target mRNA levels were normalized to β-actin expression. *p < 0.05.

siRNA-mediated *Slc3a2* knockdown inhibited mTOR1 expression, whereas pharmacological inhibition of mTOR1 with rapamycin elevated SLC3A2 expression (Fig 4A). These data suggested that SLC3A2 activation does not depend on mTOR1, but that SLC3A2 instead acts upstream of mTOR1.

Inhibition of mTOR1 led to a decrease in the UPR markers *Atf4*, *Atf6*, and *Xbp1* expression. Therefore, these genes were individually knocked down with specific siRNAs to determine the effects on mTOR1 expression. Knockdown of *Atf4*, *Atf6*, and *Xbp1* caused a decrease in mTOR1 expression following TG stimulation (Fig 4B). These data suggested that mTOR1 plays a role in the UPR.

### SLC3A2 inhibition promotes apoptosis

**Fig 5:**
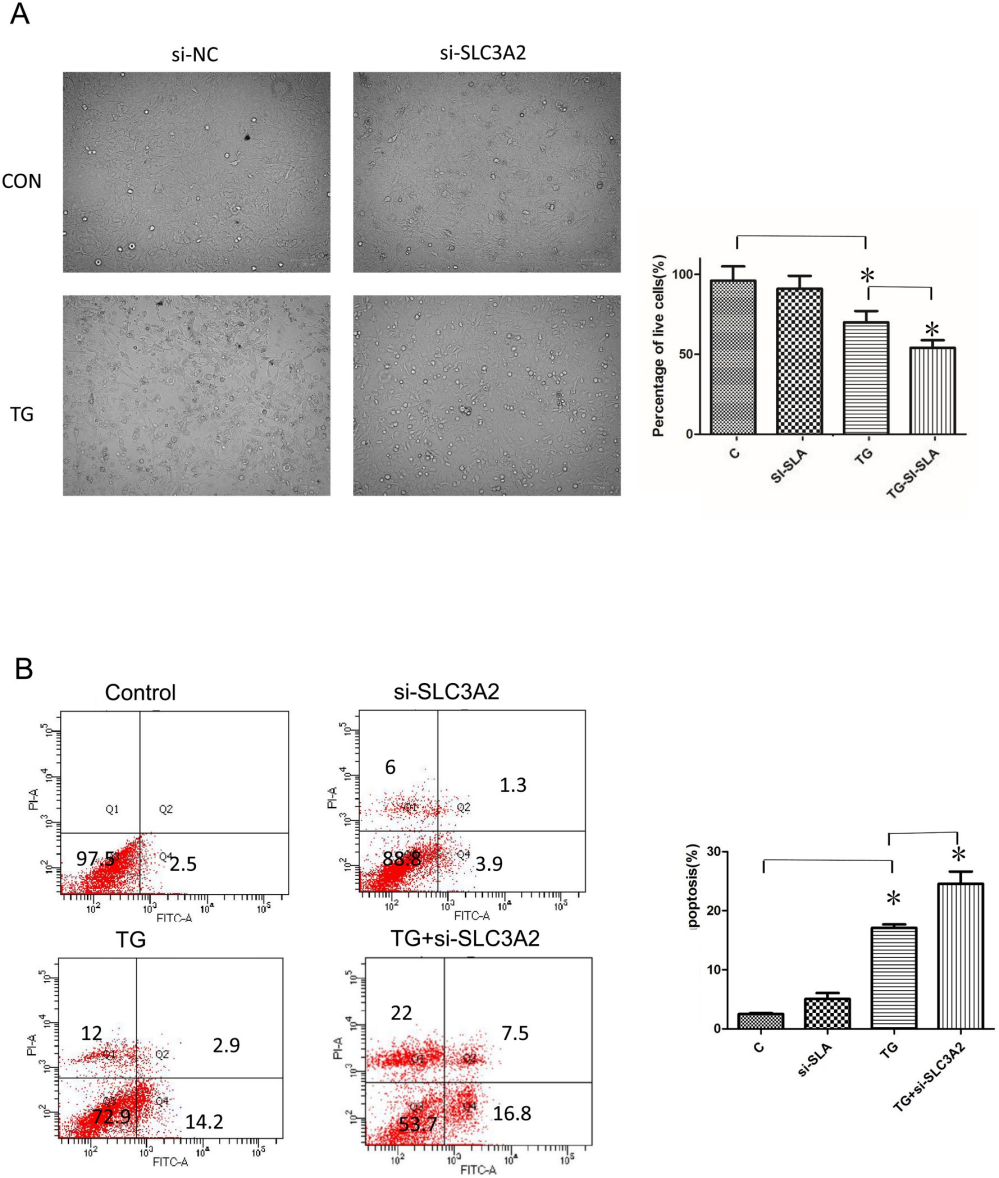
SLC3A2 knockdown increases TG-induced cardiomyocyte apoptosis. (A) Microscopic analysis of live cells following treatment with *Slc3a2* siRNA or scrambled control. (B) Flow cytometry analysis of apoptosis in TG-treated and non-treated H9C2 cells pretreated with *Slc3a2* siRNA or scrambled control. The cell population in the Q4 quadrant (Annexin V–/PI–) represents live cells, whereas the populations in the Q3 quadrant (Annexin V+/PI–) and the Q2 quadrant (Annexin V+/PI+) represent early- and late-apoptotic cells, respectively. The cell population in the Q1 quadrant (Annexin V–/PI+) represents necrotic cells. The sum of early- and late-apoptotic cells from three independent experiments is shown. ***p < 0.001 vs. control; ^###^*p* < 0.001 vs. TG treatment.

Given the link between UPR and apoptosis, the effect of SLC3A2 inhibition on ER stress-induced apoptosis was evaluated. Microscopic analysis indicated that the number of live cells was significantly decreased following treatment with TG, and a further decrease in live cells was observed following treatment with both TG and *Slc3a2* siRNA (Fig 5A). Flow cytometry analysis corroborated that apoptosis was significantly induced in cells treated with TG for 24 h, and even more so when SLC3A2 was additionally knocked down (Fig 5B). These data suggest a critical function of SLC3A2 as an anti-apoptotic factor during ER stress-induced apoptosis.

### Downstream targets of SLC3A2

**Fig 6:**
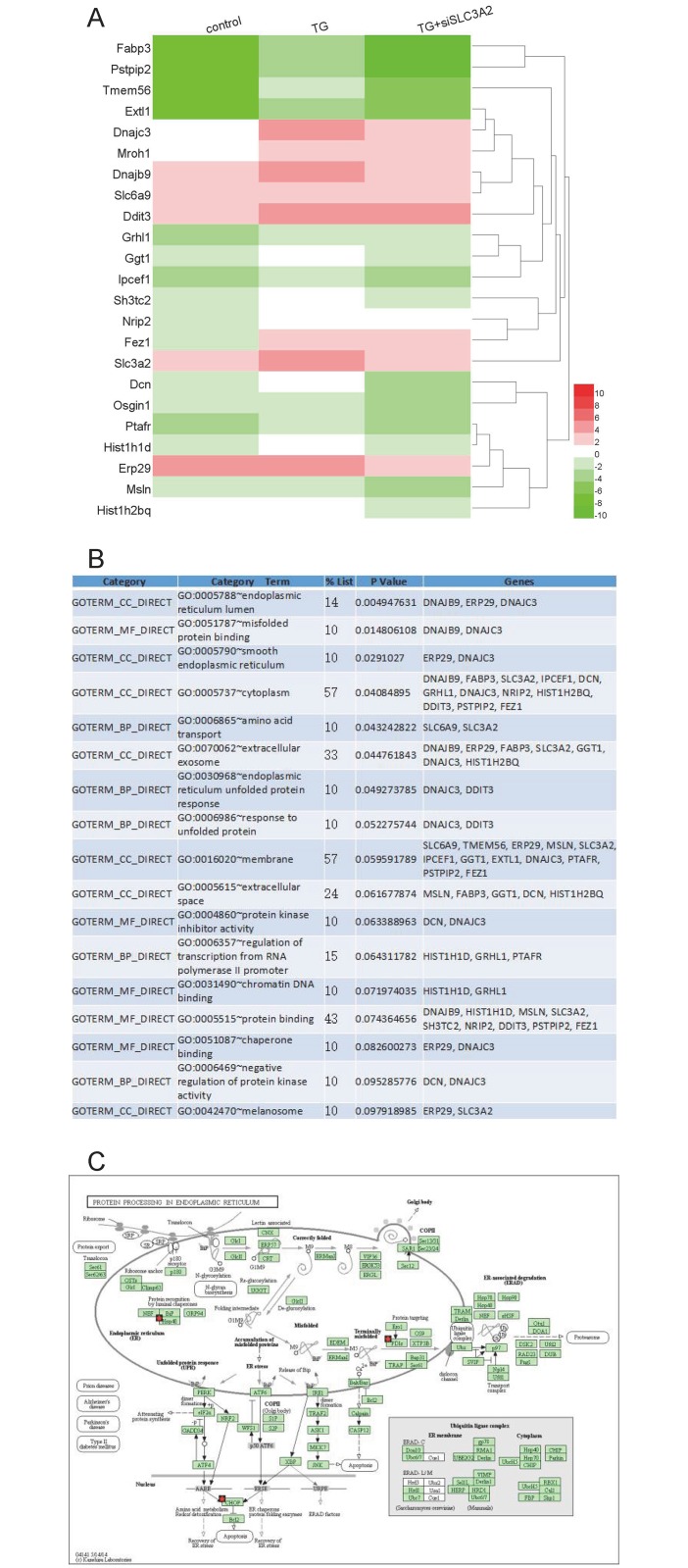
*SLC3A2* knockdown suppresses transactivation of UPR. (A) Heatmap of genes differentially expressed between control (left), scramble siRNA + TG-treated (middle), and *Slc3a2* siRNA + TG-treated (right) cells. The data are log2-transformed. Statistical criteria for differential expression were a fold change ≥ 2 and a false discovery rate (FDR) < 0.05. (B) GO analysis using DAVID for the genes shown in (A). An FDR < 0.05 was considered statistically significant. (C) Ingenuity Pathway Analysis of the genes differentially expressed in the *Slc3a2* siRNA-treated group revealing enrichment in signaling networks involved in protein processing in the ER. Genes most highly enriched in ER-related processes are indicated by a red star.

In siRNA control cells, there was a robust increase in the expression of well-characterized components of the UPR following TG treatment. Comparison of gene expression profiles between cells with and without SLC3A2 knockdown, 23 genes were found to be differentially expressed under TG-induced ER stress. This gene list included fatty acid-binding protein 3 (*Fabp3*), proline-serine-threonine phosphatase-interacting protein 2 (*Pstpip2*), transmembrane protein 56 (*Tmem56*), decorin (*Dcn*), oxidative stress-induced growth inhibitor 1 (*Osgin1*), platelet-activating factor receptor (*Ptafr*), histone cluster 1 H1 family member d (*Hist1h1d*), exostosin-like glycosyltransferase 1 (*Extl1*), mesothelin (*Msln*), Slc3a2, endoplasmic reticulum protein 29 (*Erp29*), histone cluster 1, H2bq (*Hist1h2bq*), DNA damage-inducible transcript 3 (*Ddit3*, also known as *Chop*), grainyhead-like transcription factor 1 (*Grhl1*), DNAJ heat shock protein family (*Hsp40*) member B9 (*Dnajb9*), *Hsp40* member C3 (*Dnajc3*), solute carrier family 6 member 9 (*Slc6a9*), interaction protein for cytohesin exchange factors 1 (*Ipcef1*), SH3 domain and tetratricopeptide repeats 2 (*Sh3tc2*), maestro heat-like repeat family member 1 (*Mroh1*), nuclear receptor-interacting protein 2 (*Nrip2*), gamma-glutamyltranspeptidase 1 (*Ggtl*), and fasciculation and elongation protein zeta 1 (*Fez1*) (Fig 6A). GO analysis of this set of genes revealed strong enrichment for ER lumen, misfolded protein binding, and amino acid transport (Fig 6B). We next analyzed this gene set using Ingenuity Pathway Analysis [16,17] to identify signaling networks that are specifically blocked by SLC3A2 (Fig 6C). These unbiased functional analyses revealed that the set of differentially expressed genes was largely involved in signaling pathways involved in ER protein processing.

## Discussion

ER stress plays a significant role in numerous diseases; however, the role of various proteins in the cellular response to ER stress is largely unknown. In the present study, we characterized the regulation and function of SLC3A2 in this process, based on our previous finding of its upregulation in rat cardiomyocytes during ER stress. Our data clearly indicated that SLC3A2 is an ER stress-induced protein, possibly acting upstream of ATF4, ATF6, and XBP1 in the UPR, and has anti-apoptotic effects. The role of SLC3A2 in the UPR was further highlighted by its ability to activate ATF4, ATF6, and XBP1, all of which play a critical role in the UPR signaling pathway [18]. Indeed, *Slc3a2* knockdown was sufficient to prevent *Atf4*, *Atf6*, and *Xbp1* expression. Furthermore, SLC3A2 inhibited the expression of mTOR1, which was found to interact with ATF4, ATF6, and XBP1. These data suggested that SLC3A2 functions upstream of mTOR and UPR, and SLC3A2 suppression results in a cascade response that affects multiple aspects of the UPR.

Various UPR proteins are involved in transmembrane transport [19]. As unfolded protein levels rise in the ER, cytoplasmic proteins can move toward the nucleus, where they activate the transcription of a range of target genes. Based on the results of this study, SLC3A2 protein is primarily expressed in the cytoplasm, and its localization was not affected by TG treatment. These data indicated that SLC3A2 is not translocated to the nucleus in the UPR signaling cascade.

ER stress can ultimately result in apoptosis. If ER stress is excessive and prolonged, the ER protective mechanism is not sufficient to restore ER function, and cells subsequently enter apoptosis. ATF4-CHOP pathway activation is a key event in ER stress-induced cell death. Overexpression of CHOP induces cell cycle arrest and apoptosis. Caspase-12, which is localized on the ER membrane, is specifically activated in the ER stress-related apoptosis pathway. Bcl-2, located in the ER, is also involved in the regulation of apoptosis pathways in response to ER stress [20–22]. Thus, to fully understand the role of SLC3A2 in ER stress and the UPR, we examined the influence of its knockdown on apoptosis. As expected, TG induced apoptosis, whereas SLC3A2 knockdown promoted apoptosis as assessed by flow cytometry. This was confirmed with direct microscopic observation of live and dead cells based on differential staining. These data indicated that SLC3A2 may function to protect injured cells against apoptosis. Furthermore, RNA-seq analysis demonstrated that SLC3A2 knockdown also suppressed a subset of ER stress-activated genes. GO analysis of these genes revealed that they were involved in biological processes such as ER UPR, response to unfolded protein, regulation of transcription from RNA polymerase II promoter, and negative regulation of protein kinase activity. The molecular functions assigned to the differentially expressed genes included misfolded protein binding, protein kinase inhibitor activity, chromatin DNA binding, protein binding, and chaperone binding. These data suggest that SLC3A2 acts upstream of the UPR, and SLC3A2 suppression results in a cascade response that affects multiple aspects of the UPR [23].

As SLC3A2 was initially discovered as a transporter of L-type amino acids, it was not surprising that its knockdown also affected genes involved in amino acid transport and regulation of transcription from RNA polymerase II. Based on its previously reported functions, it is possible that SLC3A2 interacts with proteins such as integrins to regulate target genes via RNA polymerase II release, or to regulate the function of protein kinases such as Src family kinase and tyrosine kinases [24–26]. While the exact function of SLC3A2 in the ER stress response requires further exploration, our findings indicate that the signature of SLC3A2-regulated genes is specifically related to protein processing in ER signaling pathways.

In conclusion, we evaluated the role of SLC3A2 in ER stress using an *in vitro* model. Our data highlight SLC3A2 as a novel ER stress-induced protein that is located in the cytoplasm and acts as an important factor in the upstream UPR. SLC3A2 also appears to protect cells against apoptosis via a CHOP-independent mechanism. To our knowledge, this is the first study to evaluate the role of SLC3A2 during ER stress. Although more studies are needed to fully elucidate the mechanism, the present study provides important insights into the role of SLC3A2 in the regulation of the ER stress-related UPR, suggesting a potential target for treatment of ER stress-related diseases.
